# Resultados clínicos finales de COVID-19 en pediatría: comparación entre pacientes inmunosuprimidos e inmunocompetentes en Colombia

**DOI:** 10.7705/biomedica.8156

**Published:** 2026-08-20

**Authors:** Paola Marsela Pérez-Camacho, Lina M. Sandoval-Calle, Inés Elvira Gómez-Hernández, Jared Vanessa Suaza-Cortés, Silvia Vanessa Rojas-Quintero, María Camila Díaz, Juan Sebastián Victoria, Laura Alejandra Torres-Cánchala, Mario Bustos, Jaime Alberto Patiño-Niño

**Affiliations:** 1 Servicio de Infectología Pediátrica, Departamento de Pediatría, Fundación Valle del Lili, Cali, Colombia Departamento de Pediatría Fundación Valle del Lili Cali Colombia; 2 Centro de Investigaciones Clínicas, Fundación Valle del Lili, Cali, Colombia Fundación Valle del Lili Cali Colombia; 3 Facultad de Ciencias de la Salud, Universidad Icesi, Cali, Colombia Universidad Icesi Facultad de Ciencias de la Salud Universidad Icesi Cali Colombia

**Keywords:** infecciones por coronavirus, SARS-CoV-2, pediatría, huésped inmunocomprometido, inmunosupresión, hospitalización, mortalidad, Coronavirus infections, SARS-CoV-2, pediatrics, immunocompromised host, immunosuppression, hospitalization, mortality

## Abstract

**Introducción.:**

Los pacientes pediátricos suelen presentar COVID-19 menos grave que los adultos; sin embargo, los resultados clínicos finales en niños inmunosuprimidos siguen siendo heterogéneos y controversiales. Algunos estudios plantean un mayor riesgo de complicaciones, mientras que otros sugieren que la inmunosupresión podría modular la respuesta inflamatoria asociada con la enfermedad grave.

**Objetivo.:**

Comparar los resultados clínicos finales de los pacientes pediátricos inmunosuprimidos e inmunocompetentes hospitalizados con COVID-19 en un centro de alta complejidad de Colombia durante la pandemia.

**Materiales y métodos.:**

Se llevó a cabo un estudio retrospectivo de cohortes que incluyó pacientes menores de 18 años hospitalizados entre el 2020 y el 2023 con COVID-19 confirmado. Se compararon las características clínicas y los resultados finales entre ambos grupos. Se usó estadística descriptiva y la prueba de ji al cuadrado o la exacta de Fisher para las variables categóricas, y la prueba t de Student o la U de MannWhitney para las variables continuas, con significancia de p < 0,05.

**Resultados.:**

Se analizaron 705 pacientes: 124 (17,6 %) inmunosuprimidos y 581 (82,4 %) inmunocompetentes. Los inmunosuprimidos tuvieron mayor mediana de edad (10 frente a 2 años) y mayor mortalidad (4,03 % frente a 2,1 %; p < 0,001). Por otro lado, los inmunocompetentes requirieron con mayor frecuencia intubación al ingreso (4,6 % frente a 0,8 %; p = 0,02) y presentaron una estancia hospitalaria más prolongada (6 frente a 5 días; p = 0,04). La falla renal fue más frecuente en los inmunosuprimidos (2,4 % frente a 0,3 %; p = 0,04).

**Conclusiones.:**

Los pacientes pediátricos inmunosuprimidos no presentaron mayor gravedad clínica aguda medida por la necesidad de soporte crítico; sin embargo, hubo mayor mortalidad. Estos resultados evidencian la variabilidad de la evolución clínica según el estado inmunológico y subrayan la necesidad de estudios adicionales para identificar los factores de riesgo y los posibles mecanismos protectores en esta población.

## Abstract (en)

**Introduction.:**

Pediatric patients generally present less severe COVID-19 compared with adults; however, clinical outcomes in immunosuppressed children remain heterogeneous and controversial. Some studies suggest a higher risk of complications, whereas others propose that immunosuppression could attenuate the exaggerated inflammatory response associated with severe disease.

**Objective.:**

To compare the clinical outcomes of immunosuppressed and immunocompetent pediatric patients hospitalized with COVID-19 in a highcomplexity center in Colombia during the pandemic.

**Materials and methods.:**

A retrospective cohort study was conducted including patients under 18 years of age hospitalized with confirmed COVID-19 between 2020 and 2023. Clinical characteristics and outcomes were compared between both groups. Descriptive statistics were used, along with chi square or Fisher's exact tests for categorical variables, and Student's t test or MannWhitney U test for continuous variables depending on their distribution, with significance set at p < 0.05.

**Results.:**

A total of 705 patients were analyzed: 124 (17.6%) immunosuppressed and 581 (82.4%) immunocompetent. Immunosuppressed patients had a higher median age (10 vs. 2 years) and higher mortality (4.03% vs. 2.1%; p < 0.001). In contrast, immunocompetent patients more frequently required intubation upon admission (4.6% vs. 0.8%; p = 0.02) and had a longer hospital stay (6 vs. 5 days; p = 0.04). Renal failure was more common among immunosuppressed patients (2.4% vs. 0.3%; p = 0.04).

**Conclusions.:**

Immunosuppressed pediatric patients did not show greater acute clinical severity in terms of critical care requirements; however, they presented higher mortality. These findings highlight the variability of outcomes according to immunological status and underscore the need for further studies to identify risk factors and potential protective mechanisms in the pediatric population with COVID-19.

La pandemia por SARS-CoV-2 (*Severe Acute Respiratory Syndrome-Coronavirus* 2) ha generado consecuencias sin precedentes a nivel mundial, no solo en términos de morbimortalidad, sino también por su impacto en los sistemas de salud, la economía global y el bienestar social. Todos los grupos etarios se han visto afectados, incluyendo la población pediátrica.

En Colombia, se han reportado más de 6,2 millones de casos confirmados, de los cuales el 11 % corresponde a población pediátrica ^1^. Aunque los niños suelen presentar formas más leves de la enfermedad en comparación con los adultos y la tasa de mortalidad en esta población es baja (0,06 % frente al 2,2 % en la población general), algunos subgrupos pediátricos podrían estar en mayor riesgo de presentar complicaciones ^1^.

Uno de estos subgrupos es el de los pacientes con inmunosupresión, quienes históricamente han sido considerados más vulnerables ante las infecciones virales -virus sincitial respiratorio, influenza o citomegalovirus-, las cuales pueden ocasionar cuadros clínicos graves en esta población ^2^. En el contexto de la COVID-19 *(Coronavirus Disease* 19), diversos estudios han sugerido que el estado de inmunosupresión se asocia con un mayor riesgo de enfermedad grave (riesgo relativo [RR] = 1,44; IC_95%_: 1,01 a 2,04) y con mayores probabilidades de desarrollar síndrome inflamatorio multisistémico en niños ^3^^,^^4^, particularmente en aquellos con inmunodeficiencias primarias, en quienes se ha reportado un riesgo relativo de 2,11 (IC_95%_: 1,05 a 4,26) ^5^.

Sin embargo, la literatura disponible reporta resultados heterogéneos y, en algunos casos, contradictorios. La asociación entre inmunosupresión, síndrome inflamatorio multisistémico y resultados finales adversos podría estar influenciada por factores subyacentes que actúan como variables de confusión, tales como la enfermedad de base, la quimioterapia o la presencia de coinfecciones, lo que puede conducir a una sobreestimación del riesgo atribuible exclusivamente a la infección por SARS-CoV-2 ^6^. En este sentido, se ha descrito que la tasa de mortalidad intrahospitalaria de los pacientes pediátricos con cáncer e infección por SARS-CoV-2 fue del 10,9 % (IC_95%_: 6,9 a 16,2), una cifra comparable a la reportada en la población general (12,5 %) ^7^, lo que sugiere que los resultados clínicos finales adversos no siempre difieren de manera constante entre las poblaciones inmunosuprimidas y las inmunocompetentes.

Por el contrario, en otros estudios se ha reportado que algunos pacientes inmunosuprimidos podrían presentar resultados clínicos similares o incluso menos graves que los inmunocompetentes, hallazgo que se ha atribuido a la respuesta inmunitaria menos exacerbada del huésped ^2^. Esta hipótesis también podría explicar por qué en determinados subgrupos se han observado menos efectos adversos a largo plazo, posiblemente relacionados con una menor carga de inflamación sistémica y neuroinflamación posterior a la infección ^8^^,^^9^.

Teniendo en cuenta los datos controvertidos y heterogéneos disponibles en la literatura, así como la limitada evidencia proveniente de cohortes pediátricas bien caracterizadas en contextos latinoamericanos, resulta importante comprender mejor los resultados clínicos finales de los niños inmunocomprometidos con infección por SARS-CoV-2. Este conocimiento es fundamental para optimizar la planificación del manejo clínico, el uso de los recursos en salud, la estimación del riesgo de complicaciones y la toma de decisiones en prevención y tratamiento.

En este contexto, el objetivo de este estudio fue describir y comparar los resultados clínicos finales de los pacientes pediátricos inmunosuprimidos e inmunocompetentes infectados por SARS-CoV-2 atendidos en un hospital de alta complejidad del suroccidente colombiano entre los años 2020 y 2023. Partimos de la hipótesis de que los pacientes inmunosuprimidos pueden presentar resultados clínicos finales similares o incluso más favorables que los inmunocompetentes, debido a una menor respuesta inflamatoria frente al virus.

## Materiales y métodos

### Diseño del estudio y participantes

Se llevó a cabo un estudio retrospectivo de cohortes en la Fundación Valle del Lili, un hospital de alta complejidad del suroccidente colombiano. Se consideraron los pacientes incluidos en el registro clínico de COVID-19 pediátrico, denominado COVID-PED, el cual fue creado en el 2020, con la recolección retrospectiva basada en la revisión de las historias clínicas; el ingreso de pacientes se actualizó cada año y la recolección se finalizó en el 2023. Dicho registro incluye a todos los pacientes menores de 18 años con diagnóstico confirmado de COVID-19 hospitalizados en la Fundación Valle del Lili con PCR o antígeno positivo para SARS-CoV-2 entre los años 2020 y 2023.

Se excluyeron del análisis del estudio a los pacientes con historias clínicas incompletas o a aquellos con estancia hospitalaria menor de 24 horas. El muestreo fue por conveniencia; se incluyeron todos los pacientes que cumplían con los criterios de inclusión durante el periodo de estudio. La evaluación de coinfecciones virales, bacterianas y fúngicas no se hizo de manera sistemática en todos los pacientes; dichas pruebas fueron solicitadas de forma individualizada por el médico tratante, según la evolución clínica y la sospecha diagnóstica durante el contexto de la pandemia por COVID-19.

### Variables medidas

Se analizaron las siguientes variables:

- epidemiológicas: la prueba para la detección de COVID-19 y el estado previo de vacunación;
- sociodemográficas: edad, sexo y estado de afiliación al sistema de salud, y
- clínicas: antecedentes patológicos y características del ingreso hospitalario, signos y síntomas.

También se consideraron datos clínicos como la estancia hospitalaria, la necesidad de hospitalización en la unidad de cuidados intensivos pediátricos y el estado vital al egreso.

Al clasificar los pacientes en dos grupos para establecer comparaciones entre los resultados finales, se consideró como «exposición» al estado inmunológico (inmunosuprimido frente a inmunocompetente) y los datos clínicos evaluados incluyeron el ingreso a la unidad de cuidados intensivos pediátricos, la necesidad de asistencia respiratoria mecánica, la estancia hospitalaria y la muerte.

Se consideró como «inmunosuprimido» a cualquier sujeto con antecedentes patológicos documentados en la historia clínica, como cáncer, trasplante de órgano sólido, trasplante de células hematopoyéticas, inmunodeficiencia, uso de medicamentos inmunosupresores, trastorno hematooncológico (talasemia), HIV, enfermedad reumática inflamatoria, enfermedad intestinal inflamatoria, enfermedad renal crónica en terapia de remplazo renal o esclerosis múltiple.

Se consideraron como «inmunocompetentes» a los sujetos que no tenían ninguno de los antecedentes descritos para el grupo de los inmunocomprometidos.

La gravedad clínica de la COVID-19 se clasificó según las categorías establecidas en el *Consenso colombiano de atención, diagnóstico y manejo de la infección por SARS-CoV-2/COVID-19,* en leve, moderada, grave y crítica, con base en los hallazgos clínicos, los signos de compromiso respiratorio y la necesidad de soporte respiratorio o hemodinámico ^10^.

### Análisis estadístico

Se hizo el análisis descriptivo de las variables. Se evaluó el tipo de distribución de las variables con la prueba de Shapiro-Wilk. Luego, las variables cuantitativas continuas con distribución normal se describieron con la media y la desviación estándar, mientras que las que tenían distribución diferente a la normal se describieron con la mediana y el rango intercuartílico. Las variables cualitativas se describieron con proporciones y porcentajes.

Se compararon los resultados clínicos finales entre los grupos, utilizando la prueba de ji al cuadrado o la exacta de Fisher para las variables categóricas, y la t de Student o la U de Mann-Whitney para las variables continuas, según correspondiera. Se consideró p < 0,05 como estadísticamente significativo. Se utilizó el paquete estadístico Stata SE™, versión 18.

### Consideraciones éticas

Este estudio obtuvo el aval del Comité de Ética de la Fundación Valle del Lili para su realización mediante el acta número 1988. Además, se considera como «sin riesgo» según la Resolución 8430 de 1993 del Ministerio de Salud de Colombia. La información fue tratada con estricta confidencialidad.

Dado el carácter retrospectivo del estudio y el uso de fuentes secundarias (bases de datos con historia clínicas), no se requirió consentimiento informado, en cumplimiento con las normas éticas vigentes.

## Resultados

Se incluyeron 705 pacientes pediátricos con diagnóstico confirmado de la COVID-19. De estos, 124 (17,6 %) tenían antecedentes de inmunosupresión y 581 (82,4 %) eran inmunocompetentes. La mediana de edad fue de 10 años (RIC: 6,5 a 12,5) en el grupo inmunosuprimido y de 2 años (RIC: 2 a 5) en el de inmunocompetentes. En ambos grupos predominó el sexo masculino: 50,8 % en los inmunosuprimidos y el 54,9 % en los inmunocompetentes (cuadro 1).

**Cuadro 1:** Características clínicas de ingreso de pacientes pediátricos con COVID-19, según su estado inmunológico (N = 705)

		Pacientes inmunocompetentes (N = 581)	Pacientes con inmunosupresión (N = 124)
		n (%)	n (%)
Edad (mediana, RIC)		2 (2-5)	10 (6,5-12,5)
Sexo			
	Masculino	319 (54,9)	63 (50,8)
	Femenino	262 (45,1)	61 (49,1)
Régimen de afiliación en salud			
	Contributivo	273 (46,9)	73 (58,8)
	Subsidiado	144 (24,7)	37 (29,8)
Medicina prepagada		127 (21,8)	8 (6,5)
	Sin información	15 (2,5)	4 (3,2)
	Vinculado	13 (2,2)	1 (0,8)
	Particular	5 (0,9)	1 (0,8)
	Extranjero	4 (0,7)	0 (0)
Clasificación clínica de la COVID-19			
	Leve	385 (66,3)	62 (50,0)
	Moderada	119 (20,5)	39 (31,9)
	Grave	48 (8,3)	5 (4,0)
	Crítica	25 (4,3)	4 (3,2)
	Sin dato	4 (0,7)	4 (3,2)
Antecedentes nutricionales			
	Desnutrición	7 (1,2)	3 (2,4)
	Obesidad	1 (0,2)	6 (4,9)
Antecedentes patológicos			
	Trastorno neurológico crónico	57 (9,8)	10 (8,1)
	Asma	54 (9,3)	11 (8,9)
	Prematuro	56 (9,6)	4 (3,2)
	Enfermedades cardiacas crónicas(incluye la cardiopatía congénita)	33 (5,7)	6 (4,8)
	Enfermedad hepática	18 (3,1)	5 (4,0)
	Enfermedad renal crónica	12 (2,1)	4 (3,2)
	Diabetes	4 (0,7)	6 (4,8)
	Esquema de vacunación completa para la edad	491 (84,1)	98 (79,0)

RIC: rango intercuartílico

La mayoría de los pacientes presentó enfermedad leve: 50 % en los inmunosuprimidos y 66,3 % en los inmunocompetentes. Los antecedentes más frecuentes fueron el asma en los inmunosuprimidos (8,9 %) y los trastornos neurológicos crónicos en los inmunocompetentes (9,8 %). La mayoría de los pacientes contaba con un esquema de vacunación completo para la edad (79 % en los inmunosuprimidos y 84,1 % en los inmunocompetentes) (cuadro 1).

Respecto a la sintomatología al ingreso, la fiebre fue el síntoma más frecuente en ambos grupos (cuadro 2). Se encontraron diferencias en la presencia de retracciones [87 (14,9 %) frente a 6 (4,8 %); p = 0,02] y somnolencia [25 (4,3 %) frente a 3 (2,4 %); p < 0,001], y fueron más frecuentes en los inmunocompetentes. También, se observaron diferencias en el uso de ayudas diagnósticas: la radiografía de tórax fue más utilizada en los inmunosuprimidos [253 (64,5 %) frente a 80 (43,5 %); p < 0,001].

**Cuadro 2:** Características clínicas al momento del diagnóstico de COVID-19 en pacientes pediátricos (N = 705)

		Pacientes inmunocompetentes (N = 581)	Pacientes con inmunosupresión (N = 124)	
		n (%)	n (%)	p
Sintomatología al ingreso				
	Fiebre	372 (64,0)	82 (66,1)	0,53
	Tos seca	205 (35,3)	34 (27,4)	0,21
	Disnea	163 (28,1)	19 (15,3)	0,39
	Retracciones	87 (14,9)	6 (4,8)	0,02
	Somnolencia	25 (4,3)	3 (2,4)	< 0,001
	Anosmia o disgeusia	0 (0)	1 (0,8)	0,20
Imagenología diagnóstica				
	Tomografía de tórax	14 (2,4)	9 (7,3)	0,08
	Radiografía de tórax	253 (43,5)	80 (64,5)	< 0,001
Hallazgo radiológico				
	Opacidades reticulares	78 (30,8)	14 (17,5)	0,03
	Consolidación	26 (10,3)	2 (2,5)	0,09
	Derrame pleural	8 (3,2)	2 (2,5)	<0,001
	Patrón en vidrio esmerilado	6 (2,4)	5 (6,3)	0,55

En cuanto a los resultados clínicos finales (cuadro 3), se evidenció una mayor frecuencia de coinfección viral en los inmunocompetentes (4,3 % frente a 2,4 %; p = 0,01), así como una mayor necesidad de intubación orotraqueal al ingreso (4,6 % frente a 0,8 %; p = 0,02). La estancia hospitalaria fue ligeramente mayor en los inmunocompetentes (mediana de 6 días; RIC 3 a 15) en comparación con los inmunosuprimidos (mediana de 5 días; RIC 3-11), con una diferencia significativa (p = 0,04). La mortalidad fue mayor en el grupo de los inmunosuprimidos (4,0 % frente a 2,1 %; p < 0,001). También, se identificó una diferencia significativa en la presencia de falla renal (2,4 % en los inmunosuprimidos frente a 0,3 % en los inmunocompetentes; p = 0,04).

**Cuadro 3:** Resultados clínicos finales de los pacientes pediátricos con COVID-19, según su estado inmunológico (N = 705)

	Pacientes inmunocompetentes (N = 581)	Pacientes con inmunosupresión (N = 124)	
	n (%)	n (%)	p
Ingreso a la UCIP	103 (17,7)	20 (16,1)	0,44
Coinfección bacteriana	63 (10,8)	18 (14,5)	0,63
Coinfección viral	25 (4,3)	3 (2,4)	0,01
Coinfección fúngica	4 (0,7)	2 (1,6)	0,16
Necesidad de intubación al ingreso	27 (4,6)	1 (0,8)	0,02
Estancia hospitalaria	6 (3-15)	5(3-11)	0,04
Choque	27 (4,6)	7 (5,6)	0,06
Fallecimiento	12 (2,1)	5 (4,0)	< 0,001
Falla renal	2 (0,3)	3 (2,4)	0,04

UCIP: unidad de cuidados intensivos pediátricos

## Discusión

Los hallazgos de este estudio aportan evidencia sobre los resultados clínicos finales de los pacientes pediátricos inmunosuprimidos infectados por SARS-CoV-2, en comparación con sus pares inmunocompetentes. Contrario a lo que podría suponerse dada la vulnerabilidad inherente del estado de inmunosupresión, tradicionalmente asociado con mayor riesgo de infecciones virales graves ^11^, nuestros resultados demuestran que los pacientes inmunosuprimidos no presentaron mayor gravedad clínica aguda durante la hospitalización -entendida como mayor frecuencia de ingreso a la unidad de cuidados intensivos pediátricos, la necesidad de asistencia respiratoria mecánica o estancia hospitalaria- en comparación con los pacientes inmunocompetentes. De hecho, algunos indicadores sugieren un curso clínico menos agresivo en el grupo de inmunosuprimidos.

La mediana de edad más alta observada en los pacientes inmunosuprimidos (10 años frente a 2 años en el grupo de inmunocompetentes) podría haber influido en la presentación clínica, ya que la edad temprana se ha asociado con mayor riesgo de formas graves de COVID-19 en la población pediátrica ^12^^,^^13^.

En el grupo de inmunosuprimidos, se evidencia una menor frecuencia de síntomas al ingreso como somnolencia y retracciones, así como menores tasas de hospitalización en la unidad de cuidados intensivos pediátricos e intubación orotraqueal, aunque estas diferencias no siempre alcanzaron significancia estadística.

No obstante, en cuanto a la mortalidad, se observó un porcentaje significativamente mayor en el grupo de inmunosuprimidos (4 %) en comparación con el grupo de inmunocompetentes (2,1 %). Este hallazgo sugiere que, si bien los pacientes inmunosuprimidos no presentaron mayor gravedad clínica aguda, sí podrían tener un mayor riesgo de muerte, posiblemente relacionado con su condición de base, la complejidad clínica subyacente o una menor reserva fisiológica.

Esta diferencia en la mortalidad contrasta con los estudios que han reportado tasas similares o incluso menores en los pacientes pediátricos inmunosuprimidos con COVID-19 ^3^^,^^14^^,^^15^, lo que pone de manifiesto la heterogeneidad de los resultados en la literatura. Estas discrepancias podrían explicarse por las variaciones en las características de la inmunosupresión, el perfil clínico de los pacientes incluidos, el contexto del sistema de salud y los criterios de hospitalización. En particular, se ha documentado que la mortalidad en esta población varía ampliamente según la región geográfica, el acceso a las unidades de cuidados intensivos y las estrategias terapéuticas disponibles ^1^^,^^16^.

Respecto al ingreso a la unidad de cuidados intensivos pediátricos, los pacientes inmunocompetentes presentaron mayor necesidad de manejo crítico en comparación con los inmunosuprimidos, aunque esta diferencia no alcanzó significancia estadística. Este hallazgo ha sido descrito previamente en las series pediátricas internacionales, en las que los pacientes inmunocomprometidos no presentaron una mayor frecuencia de ingreso a la unidad de cuidados intensivos, posiblemente relacionado con una menor respuesta inflamatoria sistémica, considerada un factor clave en la progresión hacia las formas graves de la enfermedad ^8^^,^^17^^,^^18^.

En relación con la estancia hospitalaria, los pacientes inmunocompetentes presentaron una mediana de duración mayor (6 días) frente a los inmunosuprimidos (5 días), diferencia que fue estadísticamente significativa. Este patrón se ha observado en estudios similares, en los que los pacientes inmunosuprimidos cursaron con hospitalizaciones más cortas, probablemente debido a un abordaje clínico más protocolizado y a un monitoreo más estricto asociado con su condición subyacente ^8^^,^^16^. No obstante, este hallazgo también podría estar influenciado por la menor edad media de los pacientes inmunocompetentes incluidos en el estudio, dado que los lactantes suelen requerir estancias hospitalarias más prolongadas por los criterios de observación clínica más conservadores ^17^.

En conjunto, estos resultados se alinean con datos previos que sugieren que la gravedad de la COVID-19 puede estar relacionada, en parte, con una respuesta inmunitaria exagerada del huésped ^1^^,^^19^. En los pacientes inmunosuprimidos, la atenuación de la respuesta hiperinflamatoria podría mitigar el daño mediado por el sistema inmunológico, lo cual ha sido respaldado por los estudios que describen una menor expresión de los mediadores proinflamatorios en este subgrupo, incluso en presencia de infección activa ^12^^,^^18^^-^^21^.

Una limitación metodológica relevante del estudio es la heterogeneidad clínica dentro del grupo de inmunosuprimidos, que incluyó pacientes con inmunodeficiencias primarias, enfermedades oncohematológicas, receptores de trasplantes y otros contextos de inmunosupresión iatrogénica. Esta diversidad puede introducir variabilidad en la respuesta clínica al SARS-CoV-2 y limitar la capacidad de generalización de los resultados. Además, aunque el tamaño muestral global fue adecuado, la proporción relativa de pacientes inmunosuprimidos fue baja (n = 124), lo que reduce la potencia estadística para identificar diferencias significativas en los resultados finales menos frecuentes.

Otra limitación fue el diseño retrospectivo del estudio, el cual impone restricciones inherentes, como la posibilidad de sesgo de información y de clasificación. No fue posible evaluar con precisión el estado de vacunación ni realizar la tipificación de variantes específicas del SARS-CoV-2, factores que podrían influir en los resultados clínicos finales dada la variabilidad en la transmisibilidad y virulencia entre los subtipos virales ^22^^-^^24^. Asimismo, la evaluación de coinfecciones no se hizo de manera sistemática en todos los pacientes, ya que las pruebas fueron solicitadas de forma individual por el médico tratante según la evolución clínica durante la pandemia, lo que pudo afectar su estimación.

Además, el estudio presenta un posible sesgo de selección de tipo Berkson, dado que se incluyeron exclusivamente pacientes hospitalizados con estancia mayor de 24 horas, lo que probablemente favoreció la inclusión de los casos más graves; no obstante, se empleó una definición estandarizada de inmunosupresión y una verificación rigurosa de los criterios de inclusión para mitigar estos sesgos.

A pesar de estas limitaciones, el estudio representa un aporte relevante y poco documentado en el contexto latinoamericano, y proporciona información clínica proveniente de una cohorte hospitalaria bien caracterizada de pacientes pediátricos inmunosuprimidos e inmunocompetentes.

Estos hallazgos podrían contribuir a la toma de decisiones clínicas y al diseño de estrategias diferenciadas de seguimiento, priorización y manejo de pacientes pediátricos inmunosuprimidos en casos de infección por SARS-CoV-2.
